# Cerebro-Spinal Meningitis

**Published:** 1918-11

**Authors:** 


**Cerebro-Spinal Meningitis.** By Prof. DANTE PACCHIONI. *Pathologica.* May 15, 1918.

*Pathogenesis.* Patients suffering from cerebro-spinal meningitis suffer also very often from a meningococcic rhino-pharyngitis. It has been therefore generally admitted that the meningococci invade the meninges after passing through the minute apertures of the ethmoid plate or the sphenoid bone.

Another opinion, entertained by Koch, Debove, Vincent, and the author, is that cerebro-spinal meningitis has an hematogenic origin, and this view is now generally accepted.

Following points are now established :

1) Meningococci may give rise to septicemia without causing meningitis.

2) Meningococci may be found fairly often in the patient's blood during the first days of the attack of meningitis, and even before the onset of the disease. This justifies the subcutaneous injections that are performed, together with the spinal injections, in cases of severe septicemia.

3) Lastly, why should the meningococci alone penetrate directly from the rhino-pharynx into the meninges, whereas it is known that no other bacteria does this?

It seems possible to consider that in the cerebro-spinal meningitis, a general infective process is localized in a special part of the organism.

*Mode of Spread of the Disease.* Cerebro-spinal meningitis does not seem to be very infectious. Epidemics of this disease never spread widely. However, it is known nowadays that about 15 or 20 per cent. of the healthy persons surrounding the patients are carriers of meningococci, and often suffer from a slight attack of meningococci rhino-pharyngitis.

Some authors completely deny the infectious character of meningitis, but this really seems exaggerated. Dopter and Pacchioni consider that there are no epidemics of meningitis, but epidemics of meningococcic rhino-pharyngitis, involving septicemia in some cases, this being generally accompanied by meningitis. The meningococcus should be considered as a frequent agent of rhino-pharyngitis, a possible agent of septicemia, and, when this has taken place, an almost necessary agent of meningitis.

Children are more liable than grown-up people to suffer from meningitis, for the following reasons:

They prove particularly weak against all rhino-pharynx infections.

General infections develop easily in their organism.

A certain specific immunity existing probably in grown-up people fails during childhood. Following facts seem to prove this:

1) The serum of healthy persons has rather a strong agglutinating power, considering that of patients (1/20 to 1/35.)

2) Among the soldiers, those suffering particularly from cerebro-spinal meningitis are those coming from very aerated and lonely places, where they escaped all attacks of meningococcic rhino-pharyngitis.

They failed, therefore, in acquiring the specific immunity.

Although it has been said that lice might be active agents in the spread of meningitis, it has never been controlled.

*Prophylaxis.* Presumed carriers of meningococci should be isolated for a fortnight, and special care should be taken of those with weaknesses of the rhino-pharynx.

